# Persistent bacteremia predicts poor outcomes among neutropenic patients with carbapenem-resistant gram-negative bloodstream infections receiving appropriate therapy

**DOI:** 10.1186/s12941-023-00561-7

**Published:** 2023-02-15

**Authors:** Abi Manesh Sathya Kumar, Mithun Mohan George, Kundakarla Bhanuprasad, Grace Mary John, Anu Korula, Aby Abraham, Vikram Mathews, Uday Prakash Kulkarni, Chaitra Shankar, Prasanna Samuel Premkumar, Binila Chacko, K. Subramani, George M. Varghese, V. Balaji, Biju George

**Affiliations:** 1 Department of Infectious Diseases, Christian Medical College, Vellore, Tamil Nadu India; 2 Department of Hematology, Christian Medical College, Vellore, Tamil Nadu India; 3 Department of Clinical Microbiology, Christian Medical College, Vellore, Tamil Nadu India; 4 Department of Biostatistics, Christian Medical College, Vellore, Tamil Nadu India; 5 Division of Critical Care, Christian Medical College, Vellore, Tamil Nadu India

**Keywords:** Bloodstream infections, Carbapenem resistance, Neutropenia, Persistent bacteremia

## Abstract

**Purpose:**

Identifying persistent bacteremia early in patients with neutropenia may improve outcome. This study evaluated the role of follow-up blood cultures (FUBC) positivity in predicting outcomes among patients with neutropenia and carbapenem-resistant gram-negative bloodstream infections (CRGNBSI).

**Methods:**

This retrospective cohort study conducted between December 2017 and April 2022 included patients more than 15 years old with neutropenia and CRGNBSI, who survived for ≥ 48 h, receiving appropriate antibiotic therapy and had FUBCs. Patients with polymicrobial bacteremia within 30 days were excluded. The primary outcome was 30 day mortality. Persistent bacteremia, septic shock, recovery from neutropenia, prolonged or profound neutropenia, requirement of intensive care and dialysis, and initiation of appropriate empirical therapy were also studied.

**Results:**

In our study cohort of 155 patients, the 30 day mortality rate was 47.7%. Persistent bacteremia was common in our patient cohort (43.8%). Carbapenem resistant isolates identified in the study were K.pneumoniae (80%), E.coli (12.26%), P.aeruginosa (5.16%), A.baumanii (1.94%) and E.cloacae (0.65%). The median time for sending a FUBC was 2 days (IQR, 1–3 days). Patients with persistent bacteremia had higher mortality than those without (56.76% versus 32.1%; p < 0.001). Appropriate initial empirical therapy was given to 70.9%. Recovery from neutropenia occurred in 57.4% while 25.8% had prolonged or profound neutropenia. Sixty-nine percent (107/155) had septic shock and needed intensive care; 12.2% of patients required dialysis. Non-recovery from neutropenia (aHR, 4.28; 95% CI 2.53–7.23), presence of septic shock (aHR, 4.42; 95%CI 1.47–13.28), requirement of intensive care (aHR,3.12;95%CI 1.23–7.93), and persistent bacteremia (aHR,1.74; 95%CI 1.05–2.89) significantly predicted poor outcomes in multivariable analysis.

**Conclusion:**

FUBC showing persistent bacteremia predicted poor outcomes among neutropenic patients with carbapenem-resistant gram-negative bloodstream infections (CRGNBSI) and should be routinely reported.

## Introduction

Carbapenem-resistant gram-negative bloodstream infections (CRGNBSI) have mortality rates of up to 50% and limited therapeutic options [1]. Neutropenic and other immunosuppressed patients have the poorest outcomes. Early identification of patients at risk for poor outcomes is important for optimal management and improving prognosis. However, available predictors, such as leucocyte counts, C-reactive protein (CRP), and procalcitonin, are often imprecise [2].

Persistent blood culture positivity at 48 to 72 h despite optimal therapy is an established predictor of poor outcome, death, and metastatic infection among patients with methicillin-resistant *Staphylococcus aureus* (MRSA) bacteremia and candidemia [3]. However, their role in gram-negative bacteremia is uncertain [4]. Currently, the utilization of follow-up blood cultures (FUBC) is variable across hospitals and physicians. Further, the FUBC positivity varies according to the source of bacteremia and pathogen resistance phenotype. For example, bacteremia from a urinary source is usually transient. The role of FUBC in high-risk populations, such as patients with neutropenia and carbapenem-resistant infections, is not studied so far.

As clear data is unavailable, recent guidelines from the Infectious Diseases Society of America and other international bodies do not have directives on the use of FUBC among patients with CRGNBSI [5]. This observational study was conducted to determine the role of FUBC in patients with neutropenia and CRGNBSI.

## Methods

We performed a retrospective cohort study between December 2017 and April 2022 at the Christian Medical College, Vellore, a 3000-bedded academic center, among patients with neutropenia admitted to the hematology department with CRGNBSI. FUBC was obtained as a standard of care for all neutropenic CRGNBSIs in our hospital. Patients over 15 years of age, who survived 48 h or more after the bacteremia, were included in the study. Patients with polymicrobial bacteremia and who received inappropriate therapy were excluded. Patients who did not have an FUBC were also excluded from the study. Only the first qualifying episode of carbapenem resistant gram-negative bacteremia during the duration of hospital stay of an individual patient was included. The study was approved by the Institutional Review Board and Ethics committee.

Patient data, including their comorbidities, treatment details, and outcomes, were documented from electronic medical records. All data was collected in a predesigned proforma developed for the study on an electronic data capture system. The date of positive blood culture growing carbapenem-resistant gram-negative bacteria was taken as the date of bacteremia (index date or day zero).The patient was followed up for 30 days from that date. Carbapenem resistance was defined as resistance to meropenem or imipenem according to Clinical Laboratory and Standards Institute guidelines. Persistent bacteremia was defined as a minimum of two positive blood cultures growing the same organisms with similar antibiograms at least 48 h apart during the same infectious episode. Repeat blood cultures growing different organisms were not counted as persistent bacteremia. We defined Catheter related bloodstream infection (CRBSI) bacteremia in presence of an intravascular device with > 1 positive blood culture from a peripheral vein and no other reliable sources of infection with catheter blood culture positive at least 2 h earlier than the blood culture drawn peripherally at the same time. Being neutropenic sick individuals, most patients had a central venous catheter (CVC) at the time of bacteremia and whenever feasible, the CVC was removed as standard practice in the institution. We termed the others as probable gut translocation, as it is the most common source of bacteremia as compared to CRBSIs in this population. Neutropenia in our study was defined as an absolute neutrophil count (ANC) of less than 500 cells/μL before 48 h to the date of bacteremia. Previous bacterial infections noted in the study were defined as any bacteremia or lung infections, carbapenem resistant or susceptible, isolated 30 days prior to the index date. Concomitant bacterial infections were defined as any bacteremia or lung infections, carbapenem resistant or susceptible, isolated within 30 days after the index date. Cytomegalovirus reactivation among transplant patients was reported when CMV viremia with > 1000 copies/mL was present before or after 7 days of bacterial isolation. Prolonged and profound neutropenia was documented if the neutropenic condition persisted for more than 7 days and ANC levels were ≤ 100 cells/μL. Achieving ANC levels > 500 cells/μL during the follow-up period was marked as a recovery from neutropenia. Polymyxin-based therapy referred to treatment with polymyxin, either alone or with adjunct drugs, such as trimethoprim-sulfamethoxazole or tigecycline. Ceftazidime-avibactam-based therapy included ceftazidime-avibactam along with other adjunct drugs. When both polymyxin and ceftazidime-avibactam were given with or without adjunct drugs, it was recorded as polymyxin with ceftazidime-avibactam combination therapy. We did Carba Xpert testing of carbapenem resistant isolates. Whenever feasible, ceftazidime avibactam was prescribed on the basis of Carba Xpert testing. Aztreonam was given along with ceftazidime avibactam in our study, when New Delhi Metallo Beta Lactamases (NDM) detected in the Carba Xpert test. Approval from an Infectious diseases physician was required for prescribing ceftazidime avibactam. If the patient received treatment with drugs that the organism was not resistant to in the antibiogram done on the index date, they were considered to have received appropriate empiric therapy.

The primary outcome of the study was 30 day all-cause mortality. Other outcomes of interest were persistent bacteremia, septic shock, recovery from neutropenia, prolonged and profound neutropenia, requirement of intensive care and dialysis after the index date, and initiation of appropriate empirical therapy. A telephonic follow-up to assess the primary outcome was done for participants who were discharged within the 30 day follow-up period.

Baseline characteristics for all patients enrolled are presented as means with standard deviation (SD) or medians with interquartile range (IQR) for continuous variables and as frequencies with proportions for categorical variables. Statistical comparisons between groups for categorical variables were made using Pearson’s chi-square test or Fisher's exact test as applicable while Student’s t-test and Mann–Whitney test was used for comparing continuous variables between survivors and non-survivors. Univariate analysis was performed for all risk factors predicting 30-day mortality, and hazard ratio (HR) with 95% confidence interval (CI) was determined. Multicollinearity was checked between all variables by determining variance inflation factor (VIF). We performed multivariable Cox proportional hazards regression analysis using risk factors that were significant (p < 0.05) in the univariate analysis and determined the adjusted hazards ratio (aHR) with 95% CI. Age and sex were added to the multivariable model as natural confounders. Statistical significance was considered at a p-value of < 0.05. Survival curves were plotted to visualize the 30 day all-cause mortality among patients with persistent bacteremia and without. Censoring was not done as all study participants, unless dead, were followed-up for 30 days. Log-rank test was performed to evaluate statistical differences in survival estimates between groups. We used STATA 16 (StataCorp. 2019. *Stata Statistical Software: Release 16*. College Station, TX: StataCorp LLC) for statistical analysis.

## Results

We identified 208 patients with neutropenia above 15 years of age and had CRGNBSI. Twenty-nine patients died before 48 h of bacteremia, twenty patients did not have FUBC, and four patients did not receive appropriate therapy for the bacteremia. The 155 patients who fulfilled the eligibility criteria were included in the final analysis, of whom 93(60%) were male. The mean age of patients was 36.65 years (SD, 13 years). *Klebsiella pneumoniae* species were the most commonly isolated organism (80%) followed by E.coli (12.26%), P.aeruginosa (5.16%), A.baumanii (1.94%) and E.cloacae (0.65%). All recruited patients had an underlying active hematological condition, of which, 120 (77.42%) were malignant in nature. The most common malignant conditions were acute myeloid leukemia (42.5%) and acute lymphoblastic leukemia (27.5%). Among benign conditions, aplastic anemia (71.43%) followed by myelodysplastic syndrome (17.14) were the most common. A sizable proportion of patients developed CRGNBSI after bone marrow transplant (35.4%). Among transplant patients, CMV reactivation along with CRGNBSIs was minimal (10.9%). Polymyxin E (colistin) resistance complicated more than a fifth of our patients (21.2%) (Table 1).

**Table 1:** Baseline demographic and clinical characteristics

Characteristics	Total n = 155	Survivors n = 81 (%)	Non survivors n = 74 (%)	p-value
Age (Mean ± SD)	36.65 ± 12.93	35.96 ± 13.17	37.42 ± 12.71	0.485
Gender: Male	93	46 (56.79)	47 (63.51)	0.393
Polymyxin E (colistin) resistance(n = 146)	31	14 (21.21)	17 (21.25)	0.996
Risk factors				
Hematologic condition				
Benign	35	14 (17.28)	21 (28.38)	0.307
Acute myeloid leukemia	51	31 (38.27)	20 (27.03)	
Acute lymphoblastic leukemia	33	17 (20.99)	16 (21.62)	
Other malignant conditions	36	19 (23.46)	17 (22.97)	
No recovery from neutropenia	66	15 (18.52)	51 (68.92)	< 0.001
Prolonged and profound neutropenia^a^	40	24 (29.63)	16 (21.62)	0.255
Transplant	55	33 (40.74)	22 (29.73)	0.152
Intensive care requirement	106	38 (46.91)	68 (91.89)	< 0.001
Presence of septic shock	107	37 (45.68)	70 (94.59)	< 0.001
Dialysis requirement	19	3 (3.7)	16 (21.62)	0.001
Infection details Primary organism *Klebsiella* *pneumoniae*	124	66 (81.48)	58 (78.38)	0.063
Previous bacterial infections	44	22 (27.16)	22 (29.73)	0.723
Concomitant bacterial infections	34	13 (16.05)	21 (28.38)	0.064
Cytomegalovirus reactivation ^b^ (n = 55)	6	2 (6.06)	4 (18.18)	0.158
Source of bacteremia: probable gut translocation ^c^	109	58 (71.6)	51 (68.92)	0.715
Previous exposure to polymyxin	94	46 (56.79)	48 (64.86)	0.304
Previous exposure to carbapenem	142	74(91.36)	68 (91.89)	0.905
Persistent bacteremia	68	26 (32.1)	42 (56.76)	0.002
Duration of persistent bacteremia (n=68; Median (IQR))	4 (3–7.5)	4 (3–7)	5 (3–8)	0.265
Treatment details				
Polymyxin-based therapy	75	34 (41.98)	41 (55.41)	0.299
Ceftazidime-avibactam-based	20	10(12.35)	10(13.51)	
Therapy	53	33(40.74)	20(27.03)	
Polymyxin with ceftazidime-avibactam combination alternate agents	7	4(4.94)	3(4.05)	
No appropriate empirical therapy received^d^	45	31(38.27)	14(18.92)	0.008

^a^Prolonged and profound Neutropenia is defined as absolute neutrophil count < 100 cells/mm^3^ and a duration of 7 days

^b^Cytomegalovirus reactivation- More than 1000 copies of cytomegalovirus present 7 days before or after bacterial isolation

^c^sources include pulmonary sources (6), central line related (19), Skin and soft tissue infections (14) and other or unknown sources (7)

^d^Inappropriate empiric therapy was defined as receipt of agents without documented in vitro activity

Our cohort of patients was very ill with majority having septic shock (69%), requiring intensive care (68.4%). Nineteen (12.2%) patients required dialysis, of whom sixteen died within the follow-up period. More than half of our patients (60.6%) received polymyxins in the 30 days preceding the CRGNBSI. Almost all patients (91.6%) were exposed to carbapenems over the same time period. Over a quarter of our patients (28.3%) had laboratory confirmed bacterial infection in the last 30 days prior to index date, and 21.9% developed a concomitant bacterial infection, other than the infectious episode on index date, within their follow-up period. The major source of bacteremia in our patients (70.3%) was identified as a probable gut translocation. Other sources of bacteremia included pulmonary sources/lung (3.9%), central line related (12.3%), Skin and soft tissue infections (9%) and other or unknown sources (4.5%). Source control was achieved in our patients whenever clinically applicable. Persistent bacteremia was common in our patient cohort (43.8%). The median time for sending a FUBC was 2 days (IQR, 1–3 days). The median duration of persistent bacteremia was 4 days (IQR, 3–7.5 days).

The overall mortality rate was 47.7%, and mortality was significantly higher among individuals with persistent bacteremia (56.76%, p = 0.002) than among those without (43.24%). Among our cohort of patients, 40 (25.8%) had prolonged or profound neutropenia while 89 (57.4%) recovered from neutropenia. Among patients who survived during the follow-up period, 81.48% recovered from neutropenia. About half of the patients received a polymyxin-based therapy (48.4%), and 34.2% received polymyxin and ceftazidime-avibactam combination therapy. Persistent bacteremia was seen in 39/75(52%) patients who received a colistin based therapy, 5/20(25%) patients receiving ceftazidime avibactam-based therapy and 22/53(41.51%) patients on colistin and ceftazidime avibactam combination therapy, however the difference was not statistically significant (p = 0.134). Other types of therapy included ceftazidime-avibactam-based treatment or alternate drugs, such as tigecycline. The majority of our patients received an appropriate empirical therapy (70.9%), of whom over half (54.5%) had poor outcomes at the 30-day follow-up.

**Fig. 1:**
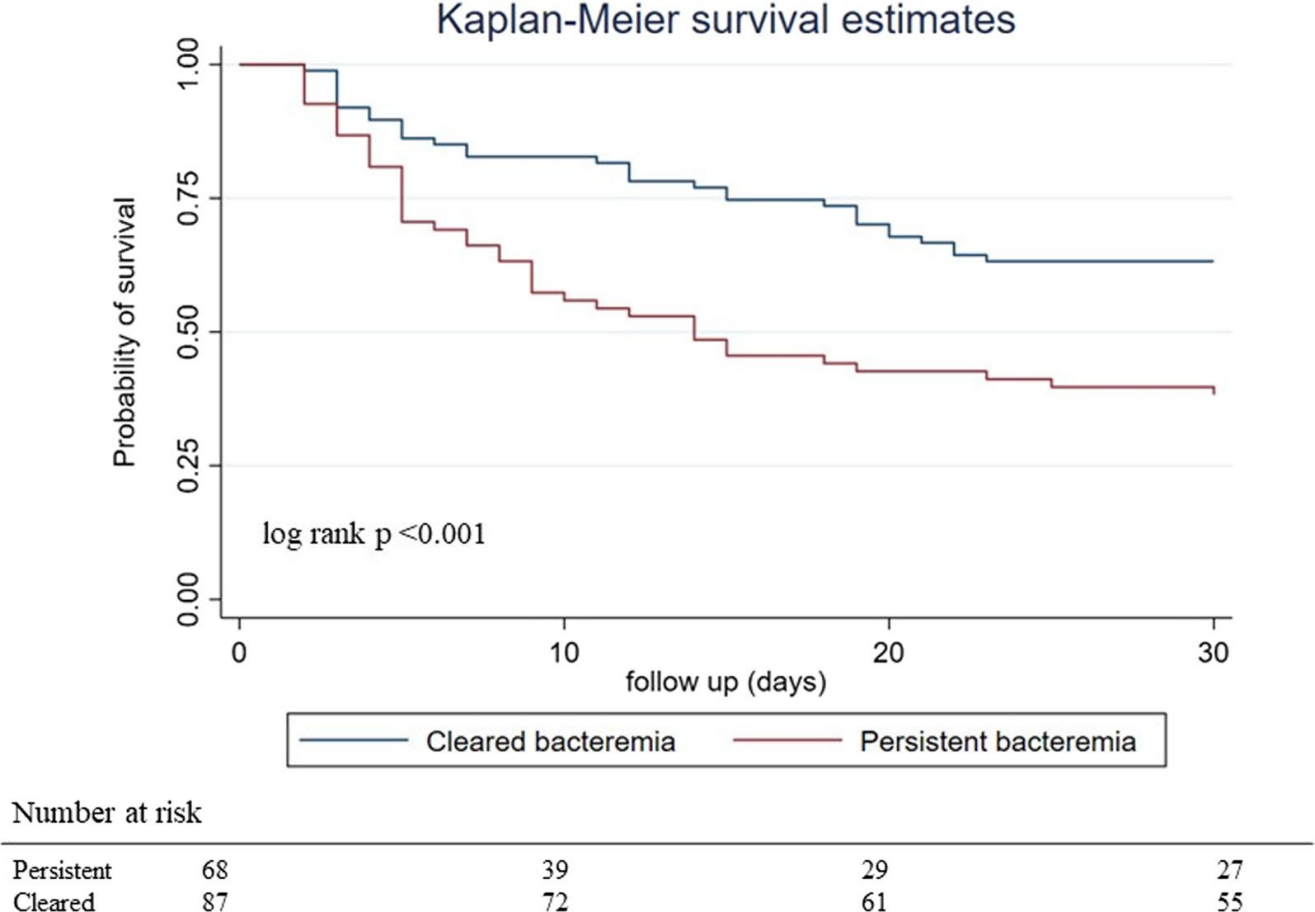
Kaplan–Meier survival curves for patients with persistent bacteremia versus cleared bacteremia

We performed univariate analysis on all patients to determine risk factors associated with 30-day mortality. Non-recovery from neutropenia (HR 5.42; 95%CI 3.28–8.93), requirement of intensive care (HR,7.84; 95%CI 3.39–18.12), septic shock (HR,12.34; 95%CI 4.5–33.88), requirement of dialysis (HR,2.75; 95%CI 1.57–4.81) and persistent bacteremia (HR,2.15; 95%CI 1.35–3.41) were significantly associated with 30-day mortality in univariate analysis. Not receiving appropriate empirical therapy did not predict mortality in our group of patients (HR 0.5; 95%CI 0.28–0.89). The mean VIF among the factors significant on univariable analysis was 1.26 with no critical correlation among the factors. When adjusted to all the significant risk factors along with age and sex as natural confounders, non-recovery from neutropenia (aHR,4.28; 95%CI, 2.53–7.23), requirement of intensive care (aHR,3.12;95%CI 1.23–7.93), presence of septic shock (aHR,4.42; 95%CI 1.47–13.28), and persistent bacteremia (aHR,1.74; 95%CI 1.05–2.89) emerged as significant associations that predicted poor outcomes in multivariable analysis (Table 2). Kaplan Meier survival curve (Fig. 1) demonstrates the survival among patients with and without persistent bacteremia in the 30 day follow-up period. Probability of survival was higher in patients without persistent bacteremia (63.2%) as compared to those with persistent bacteremia (38.2%). The difference observed in survival functions was statistically significant with p <0.001.

**Table 2:** Risk factors predicting 30-day mortality

Characteristics	Univariate analysis		Multivariable analysis	
	Hazards ratio (95% CI)	P value	Adjusted Hazards ratio (95% CI)	P value
Age	1.01 (0.99,1.02)	0.573	1 (0.98,1.02)	0.772
Male gender	1.19 (0.74,1.92)	0.463	1.4 (0.83,2.34)	0.207
Polymyxin E (colistin) resistance	0.96 (0.53,1.74)	0.904		
Hematologic condition				
Acute myeloid leukemia	0.56 (0.30,1.04)	0.065		
Acute lymphoblastic	0.73 (0.38,1.40)	0.34		
Leukemia	0.74 (0.39,1.40)	0.353		
Other malignant conditions Benign	Reference	–		
No recovery from neutropenia	5.42 (3.28,8.93)	< 0.001	**4.28 (2.53,7.23)**	** < 0.001**
Prolonged and profound neutropenia ^a^	0.65 (0.37,1.13)	0.13		
Transplant	0.67 (0.41,1.11)	0.121		
Intensive care requirement	7.84 (3.39,18.12)	< 0.001	**3.12 (1.23,7.93)**	**0.017**
Presence of septic shock	12.34 (4.5,33.88)	< 0.001	**4.42 (1.47,13.28)**	**0.008**
Dialysis requirement	2.75 (1.57,4.81)	< 0.001	1.68 (0.92,3.05)	0.091
Primary organism *Klebsiella* *pneumoniae*	0.87 (0.50,1.52)	0.625		
Previous bacterial infections	1.06 (0.64,1.75)	0.818		
Concomitant bacterial infections	1.6 (0.96,2.66)	0.069		
Cytomegalovirus reactivation^b^	2.31 (0.78,6.85)	0.132		
Source of bacteremia: Probable gut translocation^c^	0.89 (0.54,1.45)	0.639		
Previous exposure to polymyxin	1.22 (0.75,1.96)	0.421		
Previous exposure to carbapenem	0.99 (0.43,2.28)	0.983		
Persistent bacteremia	2.15 (1.35,3.41)	0.001	**1.74 (1.05,2.89)**	**0.031**
Treatment details				
Ceftazidime-avibactam-based therapy	0.82 (0.41,1.63)	0.569		
Polymyxin with Ceftazidime-avibactam combination	0.61 (0.36,1.04)	0.067		
Avibactam combination				
Alternate agents	0.75 (0.23,2.41)	0.624		
Polymyxin based therapy	Reference	–		
No appropriate empirical therapy received^d^	0.50 (0.28,0.89)	0.019	0.77 (0.42,1.41)	0.389

Bold values represents significant risk factors after multivariable analysis

^a^Prolonged and profound Neutropenia is defined as absolute neutrophil count < 100 cells/mm^3^ and a duration of 7 days

^b^Cytomegalovirus reactivation- More than 1000 copies of cytomegalovirus present 7 days before or after bacterial isolation

^c^Other sources include pulmonary sources (6), central line related (19), Skin and soft tissue infections (14) and other or unknown sources (7)

^d^Inappropriate empiric therapy was defined as receipt of agents without documented in vitro activity

## Discussion

In this study, we highlight the need for doing FUBC in patients with neutropenia and CRGNBSI. While most gram-negative bloodstream infections encountered in routine clinical practice are transient, patients with neutropenia and CRGNBSI may benefit from FUBC. Persistent bacteremia in these patients may serve as a biomarker to consider modification of treatment strategy, adequate source control, and evaluation for metastatic infections. Non-recovery of neutropenia, shock and requirement of critical care are other well known risk factors of mortality.

So far, most studies evaluating the use of FUBC in gram-negative bacteremia did not find support for their routine use [6–8]. This was because they predominantly included patients with transient bacteremia from sources like pyelonephritis, which is usually caused by organisms susceptible to most antibiotics. However, newer studies including sicker patients with resistant infections support the results of this study. Gianella et al. reported significantly higher rates of persistent bacteremia (38.5%) among patients with central line-related infections and bacteremia with carbapenem-resistant organisms [9]. However, overall mortality in their study was low, and persistent bacteremia was not associated with 30-day mortality. In another single-center large prospective study including 1702 patients with gram-negative bacteremia, two-thirds of whom had FUBC, persistent bacteremia was associated with increased mortality. Persistent bacteremia was more common with delayed initiation of effective therapy, indwelling devices, immunosuppression, and *Serratia* spp. bloodstream infections [10].

Among patients with *Staphylococcus aureus* bacteremia, persistence of blood culture positivity even after 24 h of therapy has been associated with increased mortality and metastatic complications [11]. About 40% of patients have persistent bacteremia at 24 h of effective therapy [3]. The predominant intracellular location of the pathogen, virulence markers and dysregulated host response, enables persistence, with its cell free DNA detectable in the blood even up to two weeks beyond the traditional cultures [12, 13]. Available evidence also shows reported persistent bacteremia among gram negative organisms ranging from 7.2 to 38.5% [7, 9, 10, 14, 15]. In contrast, gram negative bacteremia’s are typically extracellular, with persistence linked to persistent source, ineffective therapy, immunosuppression, and presence of carbapenem resistance [9, 10]. In our study, we excluded patients on ineffective therapy and source control was achieved as clinically indicated. Hence, we propose the persistence in our patients may be related to the neutropenia and carbapenem resistance per se.

Receipt of an appropriate empiric therapy did not show any benefit in our study cohort. Though the group had lesser mortality (18.92%, HR = 0.5; 95% CI 0.28,0.89), it did not reach significance in the multivariate analysis. Subsequent receipt of appropriate therapy may have confounded these findings and the appropriate empirical therapy group was sicker (Septic shock: 74% vs 56%). This observation has been confirmed by other studies as well [16–18]. For example, in the sub analysis of the AIDA trial data by Yael Zak- Doron et al. appropriate empirical antibiotic treatment was associated with 28 day mortality (OR, 1.372; 95% CI 1.022,1.843) in multivariable analysis [16].

This study has potential clinical implications. If the inability to clear bacteremia promptly is associated with mortality, strategies to promote clearance need to be studied. While neutropenia and immunosuppression contribute to persistent bacteremia, often they cannot be promptly reversed. Treatment modifications like switching to another agent or to combination therapies may be considered in these patients. As per the IDSA guidelines, ceftazidime avibactam, with or without aztreonam was used whenever indicated. We did not find any difference in outcomes or rates of persistent bacteremia between polymyxin and ceftazidime avibactam. Evaluation for occult metastatic infections also may be warranted in them. The ability of individual or combination antibiotics to clear the bacteremia in correlation with treatment outcomes needs to be prospectively studied. Comparing time-to-culture negativity may also aid clinicians in assessing response to treatment.

Persistent bacteremia is not routinely reported in large-scale prospective CRGNBSI studies. The PANORAMA study reported persistent bacteremia in 17% [19]. Persistent bacteremia may also serve as an important tool for assessing the efficacy of bacterial regimens in clinical trials. The duration of bacteremia may serve as an early predictor of treatment success while comparing regimens.

This study had several limitations. It is a single-center study from a country with a significant burden of CRGNBSI. Due to the retrospective design, the timing of FUBC was not standardized, although most patients had FUBCs within 96 h of bacteremia. The results may not be generalizable to patients with causes of immunosuppression other than neutropenia. A proportion of central line related bacteremia’s may be misclassified, as some patients did not have paired cultures from the central line and peripheral veins.

In summary, we propose that FUBC showing persistent bacteremia is a simple clinical tool that predicts outcomes in patients with neutropenia and CRGNBSI. Potentially, this identifies the subgroup of patients who require treatment modifications or adequate source control. FUBC may be routinely considered in these patients.

## Data Availability

All data used in the manuscript can be accessed upon request to the corresponding author.
